# The anionic biosurfactant rhamnolipid does not denature industrial enzymes

**DOI:** 10.3389/fmicb.2015.00292

**Published:** 2015-04-17

**Authors:** Jens K. Madsen, Rasmus Pihl, Anders H. Møller, Anne T. Madsen, Daniel E. Otzen, Kell K. Andersen

**Affiliations:** Interdisciplinary Nanoscience Center (iNANO), Department of Molecular Biology and Genetics, Aarhus UniversityAarhus, Denmark

**Keywords:** biosurfactant, detergent, enzyme, rhamnolipid, SDS, surfactant

## Abstract

Biosurfactants (BS) are surface-active molecules produced by microorganisms. Their combination of useful properties and sustainable production make them promising industrial alternatives to petrochemical and oleochemical surfactants. Here we compare the impact of the anionic BS rhamnolipid (RL) and the conventional/synthetic anionic surfactant sodium dodecyl sulfate (SDS) on the structure and stability of three different commercially used enzymes, namely the cellulase Carezyme® (CZ), the phospholipase Lecitase Ultra® (LT) and the α-amylase Stainzyme® (SZ). Our data reveal a fundamental difference in their mode of interaction. SDS shows great diversity of interaction toward the different enzymes. It efficiently unfolds both LT and CZ, but LT is unfolded by SDS through formation of SDS clusters on the enzyme well below the cmc, while CZ is only unfolded by bulk micelles and on average binds significantly less SDS than LT. SDS binds with even lower stoichiometry to SZ and leads to an increase in thermal stability. In contrast, RL does not affect the tertiary or secondary structure of any enzyme at room temperature, has little impact on thermal stability and only binds detectably (but at low stoichiometries) to SZ. Furthermore, all enzymes maintain activity at both monomeric and micellar concentrations of RL. We conclude that RL, despite its anionic charge, is a surfactant that does not compromise the structural integrity of industrially relevant enzymes. This makes RL a promising alternative to current synthetic anionic surfactants in a wide range of commercial applications.

## Introduction

Washing detergents are complex formulations that among many other components include surfactants and enzymes. Both surfactants and enzymes have key roles in the cleaning process. Surfactants are surface active agents that serve several roles, including reduction of surface tension, solubilization of stains and preventing redeposit. Enzymes catalyze the breakdown of difficult stains but can also act directly on fabric. The addition of enzymes to detergent formulation has made it possible to reduce washing temperatures dramatically and thereby reduce energy costs. During washing processes (and during storage in liquid detergent formulations), surfactants not only interact with stains but also with detergent enzymes. Such interaction can lead to enzyme denaturation and inactivation, which can impair washing performance. Not all enzymes are however denatured and inactivated by anionic surfactants. E.g., enzymes such as papain and pepsin (Nelson, 1971), glucose oxidase (Jones et al., 1982a) and bacterial catalase (Jones et al., 1982b) can maintain enzyme activity in the presence of anionic surfactants such as SDS. Some enzymes are even activated by surfactants as seen for lipases (Martinelle et al., 1995; Mogensen et al., 2005). In general the interactions between proteins and surfactants are many-faceted and depend on protein structure, protein surface potential, surfactant structure and charge (Otzen, 2011). Nevertheless, for optimal performance, detergents need to be formulated to maintain enzyme activity during washing.

The use of surfactants in detergents is currently dominated by surfactants produced from either non-renewable petrochemical resources or renewable plant-based resources. They are manufactured by complex chemical processes such as distillation, fractionation and hydrogenation, and are therefore considered synthetic. Continued use is however restricted by toxicity, low biodegradability, allergenicity, poor skin compatibility and strict pollution/health regulations (Lima et al., 2011). Focus is shifting to green alternatives based on sustainable production from renewable resources. Of particular interest are the so-called second generation biosurfactants (BS), i.e., surface-active compounds produced mainly by microorganisms. BS show low or no toxicity, high biodegradability and excellent surface activity at extreme pH and temperature (Edwards et al., 2003; Patel, 2003). Substitution of chemical surfactants with BS can give a 37% reduction in life-cycle CO_2_ emission, corresponding to 0.02–0.09% of total CO_2_ emission (Patel, 2003). Unlike the first generation BS produced by chemical synthesis from different sugars and lipids, second generation BS are economically increasingly attractive alternatives, in terms of cost-to-performance ratio, due to rapidly decreasing BS production costs based on production from renewable resources (Daniel et al., 1998; Daverey and Pakshirajan, 2010), yields up to 400 g/L (Franzetti et al., 2010) and rising oil prices.

One of the most promising BS is the glycolipid biosurfactant rhamnolipid (RL) from *Pseudomonas aeruginosa*. It is relevant to compare its protein interactions with that of SDS, an intensely studied model surfactant that is known to have high protein denaturation potency because of its strong binding affinity and highly charged sulfate head group (Otzen, 2011). This makes it useful in applications such as SDS-PAGE. While both SDS and RL are anionic, there are large differences in the molecular structure. SDS has a molecular weight of 265 Da (without Na counter ions) and a volume of 331.3 Å^3^ (Smith et al., 2000). In contrast mono-rhamnolipid (RL1) and di-rhamnolipid (RL2) have molecular weights of 504 and 605 Da, and volumes of 813 and 1052 Å^3^ (Chen et al., 2010), respectively. Furthermore, the anionic group of SDS is a sulfate group while that of RL is a carboxylate.

Few studies have addressed the interactions of RL with proteins. BSA has been found to bind 1–2 RL molecules which lead to increased thermal stability (Sanchez et al., 2008). At concentrations above the critical micelle concentration (cmc), RL can stabilize and facilitate folding of outer membrane proteins (Andersen and Otzen, 2014a). RL denatures both α-lactalbumin (αLA) and bovine myoglobin (Mb) (Andersen and Otzen, 2014b) but is also claimed to stabilize xylanase and—to a smaller extent—cellulases (Liu et al., 2011).

Here we systematically compare SDS and RL in their interactions with three commercial enzymes namely the cellulase Carezyme® (CZ), the phospholipase Lecitase Ultra® (LT) and the α-amylase Stainzyme® (SZ). Both CZ and SZ are commonly used in detergents. We include LT to expand the collection of industrially relevant enzymes. LT is currently not used in detergents as such, but rather for vegetable oil degumming, egg-yolk modification and lecithin hydrolysis (Bojsen et al., 2000). However, Lecitases substrates are amphiphilic just like surfactant molecules. Furthermore, during hydrolysis of substrate, LT produces and interacts with anionic free fatty acids and lysolecithin, thus making it relevant to study how LT interacts with anionic molecules and surfactants.

The 37 kDa CZ consists of a catalytic 218-residue active core (CAD) and a 38-residue cellulose binding domain (CBD). The two domains are connected by a 33 aa linker region containing 22 O-glycosylated serines and threonines and a number of prolines (Schülein, 1997), which provides great flexibility between the CAD and the CBD. While crystallization of the full enzyme has not been successful, the structure of the CAD of endoglucanase V has been solved to reveal 7 barrel-forming β-sheets and 3 α-helices, as well as a groove with two catalytically active Asp residues (Davies et al., 1993). 339-residue LT is a hybrid enzyme with aa 1–284 from the *Thermomyces lanuginosus* lipase gene and aa 285–339 from the structurally homologous *Fusarium oxysporum* phospholipase gene (Wang et al., 2011). This has led to an enzyme with the high stability of the *Thermomyces Lanuginosus* enzyme and the high activity of the *Fusarium oxysporum* enzyme (Wang et al., 2011). The α-amylase SZ originates from a *Bacillus* species.

Using spectroscopic and calorimetric approaches we show that all three enzymes interact with SDS. LT is denatured by SDS monomers, CZ is only denatured by SDS micelles, and SZ is thermally stabilized by SDS well below the cmc. However, none of the enzymes are denatured by RL and all enzymes maintain activity in the presence of both monomeric and micellar concentrations of RL. Weak interaction between RL and the enzymes LT and CZ lead to a slight thermal destabilization while SZ is thermally stabilized. This makes RL highly compatible with industrial enzymes and promising substitutions for chemical surfactants in a wide range of commercial applications.

## Materials and methods

### Materials

Tris was from AppliChem (Darmstadt, Germany), and sodium dodecyl sulfate (SDS), 4-nitrophenyl-α-D-maltohexaoside and 4-nitrophenyl butyrate was from Sigma-Aldrich (St. Louis, MO, USA). Azo-CM-Cellulose were from Megazyme International (Ireland). JBR515 rhamnolipid (RL) was provided by Jeneil Biosurfactant Company (Saukville, WI, USA) as a liquid solution consisting of 15% RL of the highest grade. JBR515 is a 1:0.35 mixture of mono-rhamnolipid (RL1) and di-rhamnolipid (RL2) with molecular weights of 504 and 650 Da, respectively. Stainzyme® plus 12 L, Carezyme® and Lecitase Ultra® were generously provided by Novozymes A/S (Bagsvaerd, Denmark) as liquid formulations. The enzymes were extensively dialyzed before experiments. LT was dialyzed against MilliQ water and SZ against 50 mM Tris pH 8. CZ required additional purification and was purified by ion-exchange chromatography on HiTrap Q sepharose FF 5 mL column (GE Healthcare, Pittsburgh, PA, USA). CZ was added to the column in 20 mM Tris pH 8.0 and washed in 20 mM Tris pH 8.0 until UV_280_ stabilized. Elution was achieved with a gradient from 0 to 500 mM NaCl in 20 mM Tris pH 8.0. Fractions with CZ were pooled and extensively dialyzed against 50 mM Tris pH 8.0. The following extinction coefficients (ε_280_) were used to determine enzyme concentration: SZ: 154.050 M^−1^ cm^−1^ (provided by Novozymes); CZ: 61.300 M^−1^ cm^−1^ and LT: 56.830 M^−1^ cm^−1^ (ε_280_ for CZ and LT calculated from the sequence).

### Determination of the critical micelle concentration and hemi micelles by pyrene fluorescence

The cmc of SDS and RL in buffer was determined by pyrene fluorescence as described in Andersen and Otzen (2009). Pyrene's fluorescence is sensitive to the environment and the ratio between the intensities of two emission peaks at 372.5 (I_1_) and 383.5 nm (I_3_) changes as pyrene partitions into surfactant micelles, making I_1_/I_3_ a good probe for the polarity of pyrene's environment (Kalyanasundaram and Thomas, 1977). Briefly, different concentrations of surfactant in buffer were prepared. After equilibration for 30 min, pyrene was added from a 100 μM stock in ethanol to a final concentration of 1 μM. Fluorescence scans were performed on a LS-55 luminescence spectrometer (Perkin-Elmer Instruments, UK), using an excitation wavelength of 335 nm, emission from 360 to 410 nm and excitation/emission slits of 5/2.5 nm. Possible complexes formed between surfactants and enzymes at concentration below the cmc were investigated by incubation of 2 μM enzyme with different concentrations of SDS or RL for 60 min before pyrene addition.

### Circular dichroism

Spectra were recorded on a JASCO J-810 spectropolarimeter (Jasco Spectroscopic Co. Ltd., Japan) equipped with a Jasco PTC-423S temperature control unit. Far-UV CD scans were recorded in the wavelength range 200–250 nm, with a bandwidth of 2 nm, a scanning speed of 50 nm/min and a response of 2 s. Measurements were conducted in a 0.1 mm quartz cuvette. Six accumulations were averaged and buffer background contributions were subtracted. Near-UV CD scans were recorded in the wavelength range 320–260 nm, with a bandwidth of 2 nm, a scanning speed of 50 nm/min and a response of 2 s. Measurements were conducted in a 1 cm quartz cuvette. Six accumulations were averaged and buffer background contributions were subtracted. Thermal scans were carried out by monitoring ellipticity at 222 nm using a temperature scan speed of 90°C/h and a data pitch collection of 0.1 nm. Measurements were conducted in a sealed 1 mm quartz cuvette. LT and SZ were measured at enzyme concentrations of 0.2 mg/mL, whereas CZ was measured at 0.4 mg/mL.

### Determination of enzyme activity

CZ: The activity of CZ was determined using Azo-CM-Cellulose as substrate. 0.2 μM CZ was incubated with 0–10 mM surfactant in 50 mM Tris pH 8.0. 2% (w/v) unbuffered substrate was mixed 1:1 (v/v) with CZ samples and mixed thoroughly. After incubation for 20 min at room temperature, 2.5 x volume of a precipitation buffer was added (300 mM sodium acetate and 20 mM zinc acetate in 75% ethanol, pH 5). After incubation for 10 min, samples were centrifuged at 2500 g in a bench top centrifuge for 10 min. Absorbance of the released product in the supernatant was measured at 590 nm with a Varioscan Platereader (Thermo Scientific, USA). Activity was normalized to the activity of CZ in buffer.

LT: The activity of LT was determined using 4-nitrophenyl butyrate (pNPB) as substrate. 0.25 μM LT was incubated with 0–10 mM surfactant in 50 mM Tris pH 8. 25 mM pNPB in 96% ethanol was mixed 1:100 (v/v) with LT samples, after which absorbance was immediately followed for several minutes at 405 nm on a Shimadzu UV-1700 PharmaSpec UV-VIS Spectrophotometer (Shimadzu Corp., Japan). Activity was determined as the slope by linear regression and normalized to the activity of LT in buffer.

SZ: The activity of SZ was determined using 4-nitrophenyl-α-D-maltohexaoside (pNPM) as substrate. 2 μM SZ was incubated with 0–10 mM surfactant in 50 mM Tris pH 8. 10 mM pNPM in 50 mM Tris pH 8 was mixed 1:10 (v/v) with SZ samples and absorbance at 405 nm was followed for several minutes using a Varioscan Platereader (Thermo Scientific, USA). Activity was determined as the slope by linear regression and normalized to the activity of SZ in buffer.

### Isothermal titration calorimetry

ITC measurements were conducted on a VP-ITC calorimeter (MicroCal, Inc., Northampton, MA). All experiments were carried out in 50 mM Tris pH 8 at 25°C, except for CZ-SDS where 23°C was used. Initial titration of CZ with SDS indicated slow denaturation kinetics around the cmc (data not shown). CZ titration parameters were therefore optimized with regards to temperature and spacing time between injections. A temperature of 23°C and a spacing of 900 s gave satisfactory and reproducible results. The reference cell was filled with water and the ITC parameters adjusted to optimize the different experiments to account for kinetics. CZ was investigated in a concentration range of 0–3 mg/mL, LT in a range of 0–4 mg/mL and SZ in a range of 0–4 mg/mL. SDS injections were performed with different conditions for optimized SDS concentration, spacing and temperature. SDS-LT experiments were performed with 100 mM SDS and a spacing of 600 s, SDS-CZ with 40 mM SDS at a spacing of 900 s and SDS-SZ with 4 mM SDS at a spacing of 1000 s. In RL experiments, parameters were varied to a lesser degree, all being performed with 25 mM RL injectant with a spacing of 300 s for both LT and SZ and 450 s for CZ. The heat signals were integrated using the Origin software supplied by MicroCal, Inc. To calculate the binding stoichiometry, enzyme dilution during ITC analysis was taken into account.

## Results

### The enzymes vary in their level of interaction with SDS and RL below the cmc

Proteins may interact with both monomeric or micellar surfactant. We therefore determined the exact critical micelle concentration of SDS and RL in buffer using the hydrophobic probe pyrene whose fluorescence is sensitive to environmental factors.

**Figure 1:**
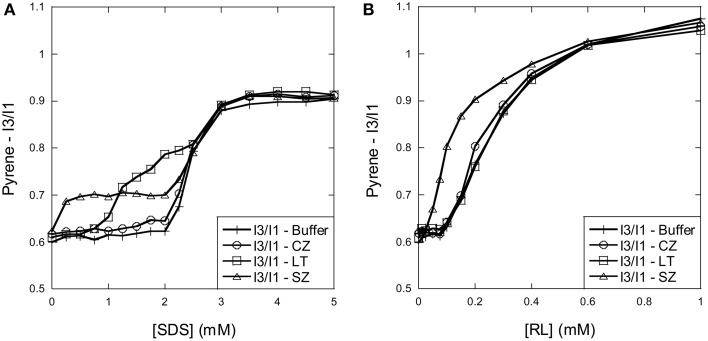
**Using pyrene fluorescence to determine SDS and RL cmc and cluster formation in the presence of the three enzymes. (A)** SDS: The I3/I1 ratio of pyrene changes around 2–3 mM SDS in the absence of enzymes. In the presence of SZ and LT the I_3_/I_1_ ratio already starts to rise at 0.25 and 1 mM SDS, respectively, indicating formation of SDS micellar clusters on the enzyme surface below the cmc. **(B)** RL: The I3/I1 ratio of pyrene changes around 0.1–1 mM RL in the absence of enzymes. The increase of the I_3_/I_1_ ratio increases already at 0.05 mM in the presence of SZ suggests that RL forms micellar clusters on the surface of SZ below the cmc.

The cmc of SDS is around 7 mM in water but reduces with increasing ionic strength (Jönsson et al., 1998). Incubation of pyrene with increasing concentrations of SDS reveals a systematic development in the I_3_/I_1_ fluorescence ratio with increasing SDS concentration: the ratio is stable around 0.6 between 0 and ~2 mM SDS where after it increases to reach a plateau of 0.9 at ~3 mM SDS (Figure 1A). This indicates that micelles are formed in solution around 2–3 mM SDS. The three enzymes all behaved in different ways in the pyrene model system. CZ did not change the titration pattern (Figure 1A), indicating that SDS does not form micellar structures on CZ below the cmc. In contrast, both LT and SZ lead to change in pyrene fluorescence below the cmc. For SZ the I_3_/I_1_ ratio increased from 0.6 to a plateau around 0.7 already at 0.25 mM SDS, indicating interactions at very low SDS concentrations. The ratio then merged with the protein-free SDS curve around the cmc. For LT the ratio was stable at 0.6 until ~0.75 mM SDS where after the I_3_/I_1_ ratio steadily increased with increasing SDS concentration, but only merged with the protein-free sample at a ratio of ~0.8, in the middle of the transition region.

RL has a much lower cmc than SDS. In buffer the I_3_/I_1_ ratio remains stable at 0.6 until 0.1 mM RL, after which it increases to ~1.05 at 1 mM RL (Figure 1B). This concentration range is in good agreement with other studies which report cmc values of rhamnolipid mixtures between 0.1 and 1 mM (Sanchez et al., 2007; Chen et al., 2010). Neither CZ nor LT affected the pyrene fluorescence pattern, indicating that the enzymes do not aid RL micelle formation below the cmc. However, for SZ the I_3_/I_1_ ratio already rises abruptly from ~0.05 mM RL, and only merges with the protein-free sample around 0.6 mM RL. This indicates that SZ interacts with monomeric RL, leading to micelle-like structures on the surface of SZ.

### Investigation of enzyme secondary- and tertiary structure by far-UV and Near-UV CD

To investigate whether surfactant clustering on the enzymes below the cmc is accompanied by denaturation, we used far- and near-UV circular dichroism to analyze how the surfactants affected enzyme secondary and tertiary structure.

**Figure 2:**
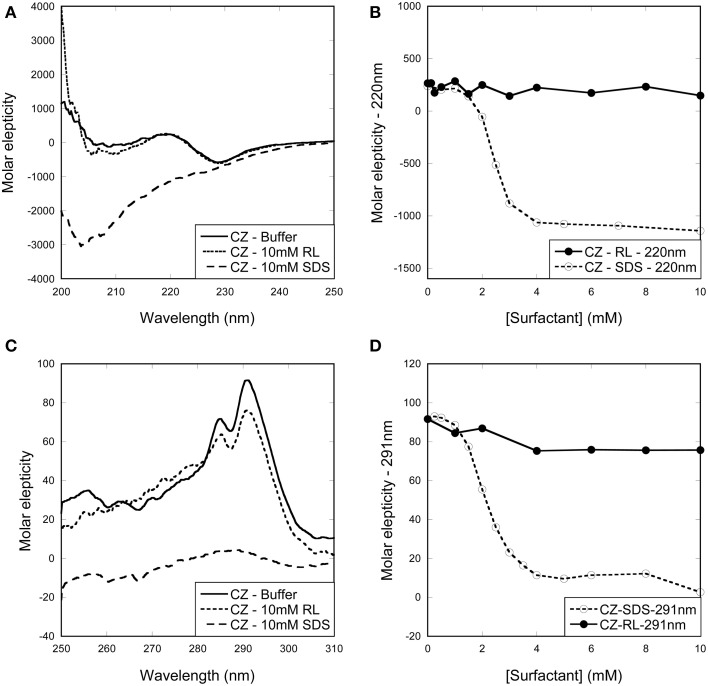
**Change in (A,B) secondary and (C,D) tertiary structure of CZ with increasing surfactant concentration. (A)** Far-UV and **(C)** near-UV CD spectra of CZ in buffer and in the presence of surfactants. Spectra of CZ in buffer and with RL are essentially identical, while SDS induces changes in both secondary and tertiary structure. Changes in the ellipticity at **(B)** 220 nm and **(D)** 291 nm reveal a structural change around 2 mM SDS. This coincides with the formation of micelles in the bulk phase.

Far-UV CD spectra of CZ in buffer show a local minimum and maximum at 230 and 220 nm, respectively (Figure 2A). Titration with RL did not lead to any change in the spectra indicating that neither monomeric or micellar RL denature CZ. In contrast, super-cmc SDS concentrations led to large spectral change; plotting ellipticity at 220 nm as a function of SDS show that the change in secondary structure occurs around the cmc (Figure 2B). These conclusions were reinforced by near-UV CD spectra (Figure 2C). Titration of CZ with RL did not lead to any change in spectra while titration with SDS led to disappearance of the two local maxima at ~285 and 295 nm around the cmc region (Figure 2D). Thus, CZ is only denatured by SDS micelles formed in the bulk phase.

**Figure 3:**
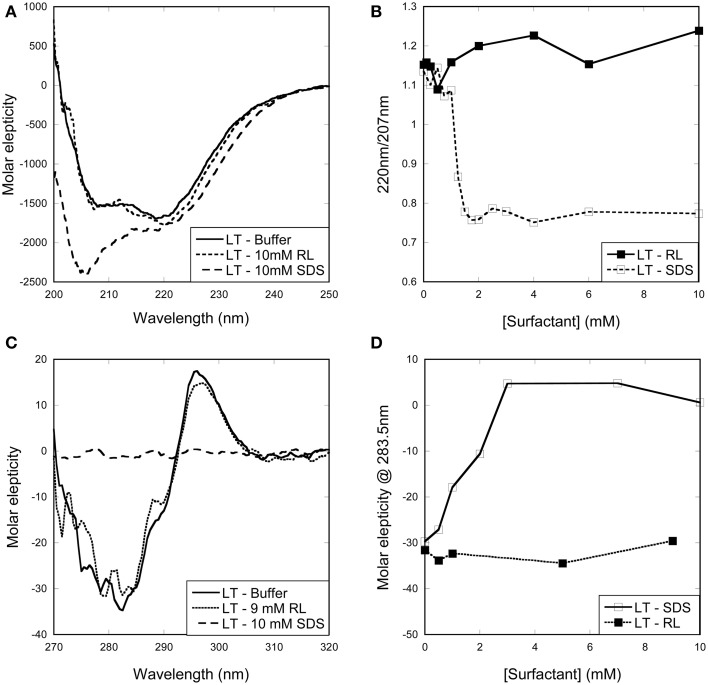
**Change in (A,B) secondary and (C,D) tertiary structure of LT with increasing surfactant concentration. (A)** Far-UV and **(C)** near-UV CD spectra of LT in buffer and in the presence of surfactants. Spectra of LT in buffer and with RL are essentially identical, while SDS induces changes in both secondary and tertiary structure. **(B)** Changes in the ellipticity ratio 220/207 nm reveal a structural change at 1 mM, i.e., below the cmc. **(D)** Changes in the ellipticity at 283.5 nm confirm that a structural change is induced by SDS below its cmc.

In the case of LT, the enzyme preserves native tertiary and secondary structure in the presence of RL monomers and micelles, while SDS leads to large changes in both secondary and tertiary structure (Figures 3A,C). The change in both secondary and tertiary structure is induced already around 1 mM SDS and the transition is complete around 2 mM SDS (Figures 3B,D). This indicates that LT is denatured below the cmc and therefore by SDS monomers.

**Figure 4:**
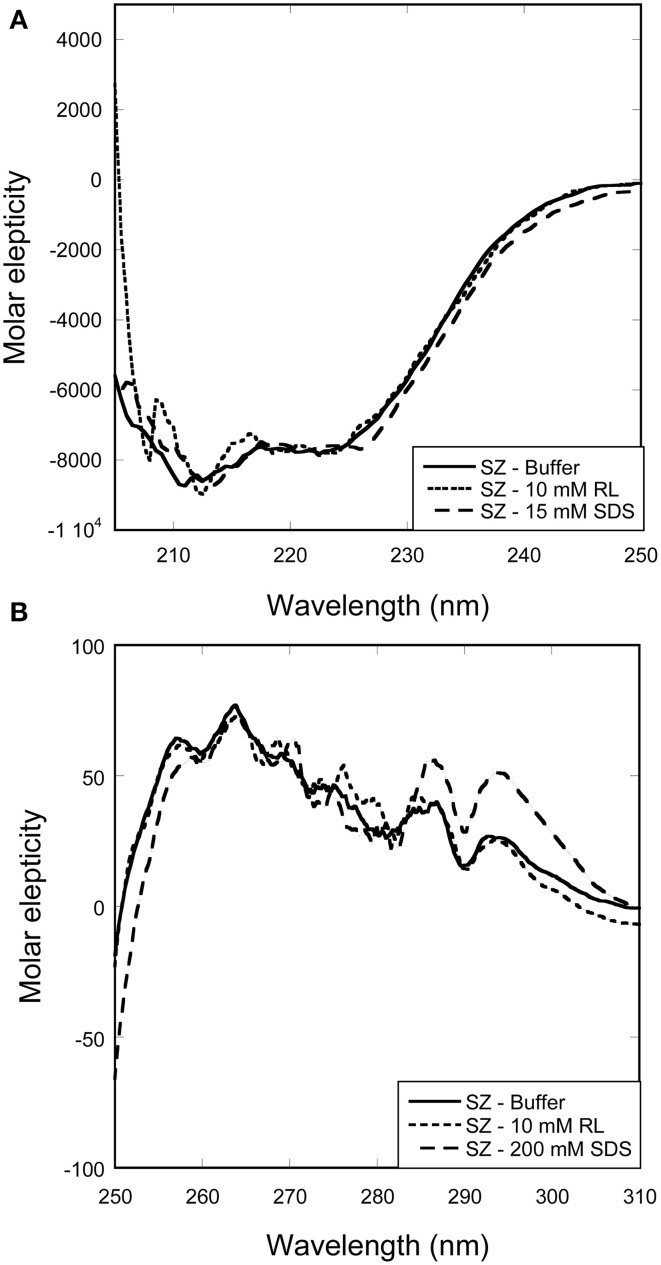
**Change in secondary and tertiary structure of SZ with increasing surfactant concentration**. Overlapping spectra shows that neither RL nor SDS induces changes in the **(A)** secondary or **(B)** tertiary structure of SZ.

Titrations of SZ with RL or SDS did not lead to any changes in either far-UV or near-UV CD spectra (Figure 4). This indicates that SZ is a stable enzyme that preserves its native structure in the presence of both SDS and RL.

### Thermal stability of all enzymes are affected less by RL than by SDS

**Figure 5:**
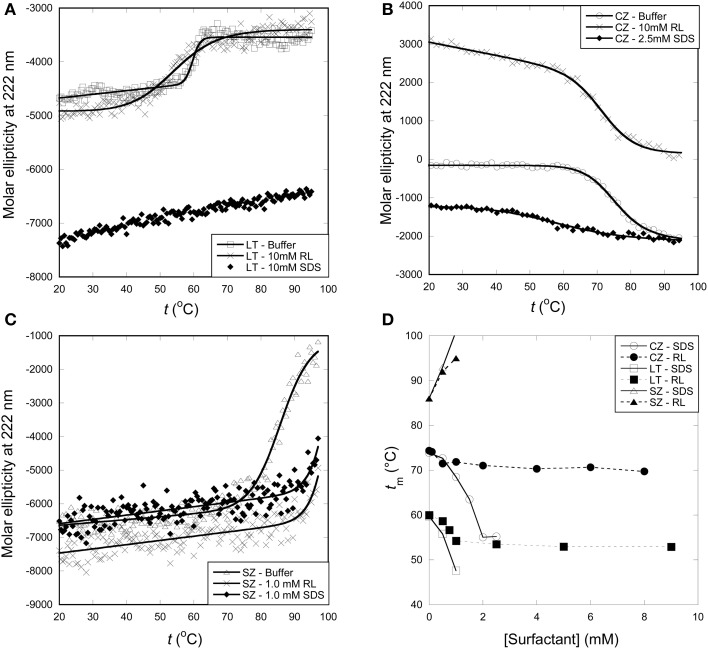
**Thermal stability of enzymes with surfactants monitored by far-UV CD thermal scans at 222 nm. (A)** *t*_m_ of LT is reduced from 60°C in buffer to ~54°C around the cmc of RL. Higher concentrations of RL do not reduce t_m_ any further, while SDS progressively lowered the *t*_m_ and no thermal transition was observed above ~1 mM SDS. **(B)** *t*_m_ of CZ is lowered by RL by a few degrees while SDS lowers the *t*_m_ from 74°C to ~58°C at 2.5 mM SDS. A thermal transition was not observed >2.5 mM SDS. **(C)** *t*_m_ of SZ was increased by both RL and SDS. **(D)** Change in enzyme thermal stability with increasing concentrations of surfactants.

Pyrene fluorescence and CD indicate that at room temperature, none of the enzymes are denatured by RL, while LT is denatured by SDS monomers and CZ by SDS micelles. Pyrene investigations indicate that SZ interact with both RL and SDS monomers, but the interactions do not lead to enzyme denaturation. To determine how SDS and RL influenced enzyme stability at elevated temperatures, we subjected all 3 enzymes to thermal scans monitored by far-UV CD at 222 nm. In 50 mM Tris pH 8 and in the absence of surfactant, melting temperatures were 86, 74, and 60°C for SZ, CZ, and LT, respectively (Figures 5A–C). As summarized in Figure 5D, both SDS and RL shifted SZ's unfolding curve to higher temperatures, indicating that the surfactants bind to the native state of SZ and actually stabilize it against denaturation. This is in excellent agreement with the observation that SZ interact with both monomeric SDS and RL but is not denatured by either surfactant. SZ's high intrinsic thermal stability made it difficult to determine melting temperatures (*t*_m_) in the presence of surfactants, since unfolding was incomplete at 95°C (the temperature limit in the experiment). Therefore it is not possible to determine the exact *t*_m_ at concentrations above 1 mM surfactant.

1 mM RL (a concentration where RL micelles are present as the majority species) reduced LT's *t*_m_ by about 7°C, but higher concentrations did not lower the *t*_m_ further. In contrast, SDS continuously lowered *t*_m_ without reaching a plateau *t*_m_-value; 1 mM SDS reduced *t*_m_ to 48°C and at higher concentration no thermal unfolding could be observed. We conclude that >1 mM SDS, LT is already denatured at room temperature, which is consistent with investigations of the enzymes' secondary and tertiary structure.

A similar pattern was seen for CZ: RL lowered *t*_m_ only slightly from 74°C in the absence of surfactant to ~70°C at 1 mM RL and above. With SDS, the thermal transition decreased steadily from 74 to ~45°C at 2 mM SDS and no thermal transition was seen at higher SDS concentrations.

### Enzyme activity is correlated to surfactant induced structural change

**Figure 6:**
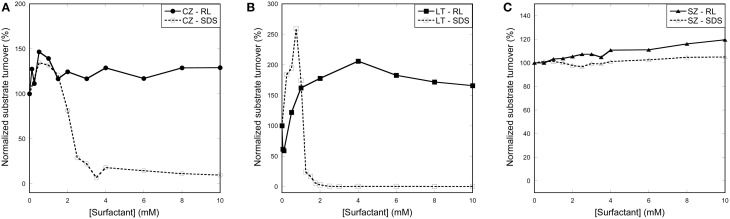
**Activity of enzymes with increasing concentration of surfactant. (A)** CZ activity declines steeply around 2 mM SDS while RL has little effect on activity. **(B)** LT activity is increased between 0 and 1 mM SDS where after it declines to ~0% between 1 and 2 mM SDS. LT activity is decreased at low RL concentration but increases at concentrations above the cmc. **(C)** SZ activity is only slightly affected by both SDS and RL.

To further consolidate our understanding of the difference in how the two anionic surfactants interact with enzymes, we monitored the enzymatic stabilities of all three enzymes as a function of surfactant concentration. Our results (Figures 6A–C) nicely corroborate the stability data. The activity of CZ in SDS increases slightly at low SDS concentrations, but then starts to decline steeply around 2 mM SDS (Figure 6A), exactly the same concentration range where our CD data indicate onset of unfolding. Activity is retained in RL at all concentrations, consistent with our CD data.

LT activity increases at low SDS concentrations but is reduced to 0.1–0.4% between 1 and 2 mM SDS (Figure 6B). This correlates well with CD determined unfolding which occurs between 1 and 1.75 mM SDS. RL shows a more complex effect on LT activity; a 40% reduction in activity at very low RL concentrations (50–100 μM) is followed by an increase in activity to 150–200% at concentrations above the cmc (1–10 mM). We have no simple explanation for this reduction and subsequent recovery in activity though it may be related to competition with the hydrophobic substrate for the active site or other interactions between substrate and RL. However, we note that RL has no adverse effect on activity above 0.1 mM, consistent with its lack of effect on LT structure.

Finally, SZ clearly retains activity at all tested SDS and RL concentrations (Figure 6C), which is completely consistent with the lack of unfolding in either SDS or RL.

### ITC reveals major differences in the binding stoichiometry of surfactants to enzymes

As a further aid to explain the (de)stabilizing effects of surfactants on enzymes, we used ITC to resolve the binding stoichiometry. ITC provides valuable information about the thermodynamics and stoichiometry of binding as shown in several studies (Nielsen et al., 2005, 2007; Bagger et al., 2007; Andersen et al., 2008, 2009; Otzen et al., 2009). Different protein concentrations of the three enzymes were therefore subjected to titrations with SDS and RL and the recorded heat flow was accordingly analyzed.

**Figure 7:**
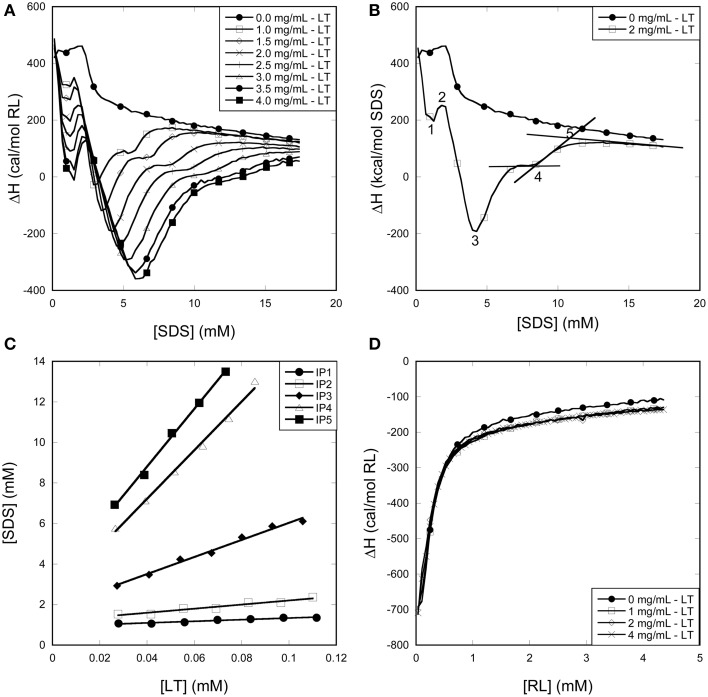
**Using ITC to determine the binding stoichiometry of surfactants to LT. (A)** Enthalpograms for the titration of SDS into LT. **(B)** Representative enthalpograms which illustrate inflection points used to calculate binding numbers. **(C)** SDS inflection points plotted as a function of LT concentration. The linear fit to Equation (1) provides binding numbers (see text). **(D)** Titration of RL into LT did not show any significant effect of the presence of the enzyme.

Dilution of micellar SDS into buffer resulted in an endothermic signal at low SDS concentrations as a result of the dissociation of SDS micelles (Figure 7A). Above 2 mM SDS there is a decrease in the endothermic signal which levels out from around 3 mM SDS, indicating that no demicellization occurs. Thus, ITC concurs with pyrene fluorescence in establishing SDS's cmc to be around 2–3 mM in our buffer system.

#### Titration of SDS and RL into LT

LT titrations with SDS at 25°C results in a number of reproducible transitions that shift to higher SDS concentrations with increasing LT concentrations (Figure 7A). Unlike SDS titrations into buffer, these titrations show an exothermic minimum (although the net signal is overall endothermic) <2 mM SDS, showing (like pyrene data) that LT interacts with monomeric SDS. Above 2 mM SDS, further interactions between SDS and LT result in a second and much larger exothermic minimum, after which a steady plateau region is reached between 5 and 10 mM SDS increasing with increasing protein concentration. Finally the signal merges with the protein-free signal from > ~10 mM SDS, indicating that no more interaction between SDS and LT occur. To obtain the stoichiometry of binding, we define five characteristic inflection points (IP) in the LT/SDS enthalpogram which systematically increase with protein concentration (Figure 7B), and plot the SDS concentration at the different inflection points as a function of LT concentration (Figure 7C). The binding stoichiometry may be derived using the following mass balance:

$${\left[S\right]}_{Total}={\left[S\right]}_{Free}+N[Protein]$$

where *N* is the number of surfactant molecules bound per protein and [*S*]_*free*_ is the concentration of surfactant that is not bound to protein. Results are summarized in Table 1.

**Table 1:** **Binding parameters derived from ITC data^a^**.

**Enzyme**	**Inflection point**	**[Surf]_free_ (mM)^b^**	**Surf. pr. Enzyme^b^**	**Amino acids per Surf**.	**g Surf/g Enzyme^c^**
LT	LT-SDS-1	0.93 ± 0.03	4.0 ± 0.4	85.5	0.03 ± 0.00
	LT-SDS-2	1.19 ± 0.08	10.1 ± 1.1	33.5	0.08 ± 0.01
	LT-SDS-3	1.82 ± 0.15	42.1 ± 2.0	8.0	0.34 ± 0.01
	LT-SDS-4	2.42 ± 0.29	120.0 ± 4.9	2.8	0.98 ± 0.02
	LT-SDS-5	3.10 ± 0.25	142.6 ± 4.7	2.4	1.16 ± 0.02
SZ	SZ-SDS-1	0.07 ± 0.00	10.5 ± 0.3	46	0.05 ± 0.00
	SZ-RL-1	0.27 ± 0.03	11.1 ± 0.6	44	0.11 ± 0.01
CZ	CZ-SDS-1	2.03 ± 0.06	22.9 ± 1.8	12.6	0.18 ± 0.01^d^
					0.22 ± 0.02^e^
	CZ-SDS-2	2.52 ± 0.03	34.2 ± 0.9	8.5	0.26 ± 0.01^d^
					0.33 ± 0.01^e^

a All experiments done in 50 mM Tris pH 8 at 25°C, except CZ-SDS where 23°C was optimal.

b Data based on fits in Figures 7C, 8C,F, 9C.

c Errors calculated based on errors in RL:protein stoichiometry (column 4).

d Based on the mass of glycosylated CZ (~37 kDa).

e Based on the mass of non-glycosylated CZ (~30 kDa).

The position of IP1 was determined by fitting the points around the minimum to a 2nd order polynomial and deriving the position of the minimum from the fitting parameters, leading to a satisfactory linear fit, with a binding number of ~4 and a free [SDS] of 0.93 mM. Clearly LT binds significant amount of SDS well below the cmc. These numbers rise as we progress through the different IPs, until at we reach IP5, where LT is fully saturated with SDS, and the 339-residue enzyme binds 143 ± 5 SDS molecules. This corresponds to one SDS molecule pr 2.4 amino acids, i.e., 1.16 g SDS/g LT. Globular proteins typically bind 1.4 g SDS/g protein (Reynolds and Tanford, 1970), though values of 1.5–2 g SDS/g protein have also been reported (Tanford, 1980). The presence of disulfide bonds can reduce this binding ratio by up to a factor of 2 (Pitt-Rivers and Impiombato, 1968), which is certainly compatible with our data in view of LT's 3 intact disulfide bonds.

Importantly, titration of RL into LT showed no difference compared to RL titration into buffer (Figure 7D). We conclude that in contrast to SDS, we are not able to detect significant levels of interaction of RL with LT at room temperature. The minor reduction in LT thermal stability by RL (Figure 5) may reflect a small degree of binding to the denatured state at elevated temperatures, displacing the equilibrium toward the denatured state.

#### Titration of SDS and RL into SZ

The SZ/SDS enthalpogram showed an exotermic signal at very low SDS concentrations (Figure 8A) with a minimum region that widened with increasing SZ concentrations (Figure 8A). It was not possible to increase the SZ concentration beyond 1 mg/mL because of visible precipitation in the presence of SDS, possibly due to neutralization of exposed positively charged residues by SDS. Subsequently the signal merged with the signal for SDS titration into buffer at concentrations (0.2–0.4 mM) well below the cmc. Within error, SZ did not change the enthalpic signals occurring around the cmc (data not shown). The position of IP1 (Figure 8B) increased linearly with [SZ] to yield a binding number of 10 SDS per protein (Figure 8C and Table 1), consistent with the thermal stabilization at very low SDS concentrations (Figure 5). This very low binding level, corresponding to one SDS molecule per 46 amino acids, is also consistent with CD investigations that showed that SZ native and tertiary structure is preserved even in the presence of micellar concentrations of SDS. The low binding number also explains the very weak effect of SZ on the enthalpogram of SDS around the cmc.

**Figure 8:**
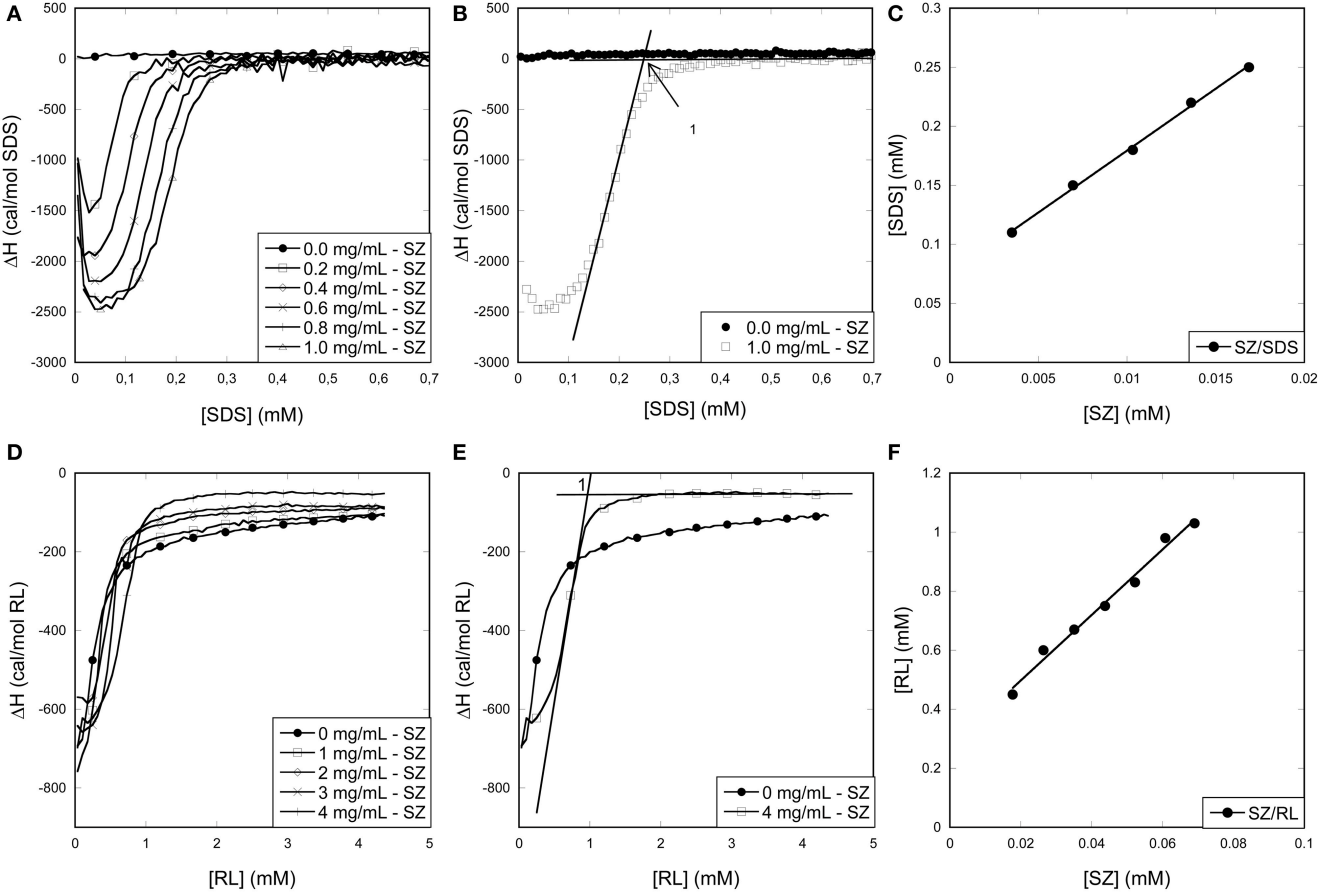
**Using ITC to determine the binding stoichiometry of surfactants to SZ**. Data for titration of **(A–C)** SDS or **(D–F)** RL into SZ. **(A,D)**: Raw enthalpograms of titration. **(B,E)**: Representative enthalpograms highlighting inflection points used to calculate binding numbers. **(C,F)**: Inflection points plotted as a function of SZ concentration. The linear fit provides binding numbers (see text).

SZ titrations with RL resulted in enthalpograms which only differed slightly from RL titration into buffer (Figure 8D). There was however a small but systematic increase toward higher RL concentrations with increasing SZ concentration. We define IP1 as the concentration where the signal levels off (Figure 8E); IP1 scales linearly with [SZ], leading to a binding number of 10 RL molecules per SZ (Figure 8F), identical to the binding number of SDS. The higher molecular weight of RL means that SZ binds almost twice as much RL by weight as SDS. However, at IP1, [RL]_free_ is 0.27 mM, which is much larger than [SDS]_free_ = 0.07 mM, indicating weaker binding overall (since a higher concentration of RL is required to reach a binding number of 10).

#### Titration of SDS and RL into CZ

**Figure 9:**
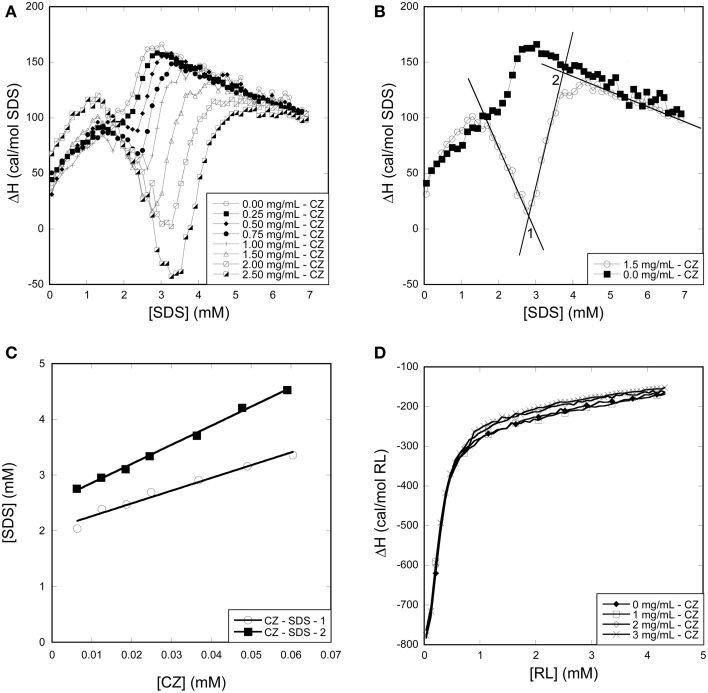
**Using ITC to determine the binding stoichiometry of surfactants to CZ. (A)** Enthalpograms for the titration of SDS into CZ. **(B) Representative** enthalpograms which illustrate inflection points used to calculate binding numbers. **(C)** SDS Inflection points plotted as a function of CZ concentration. The linear fit provides binding numbers (see text). **(D)** Titration of RL into CZ did not show any significant effect of the presence of the enzyme.

All CZ titrations with SDS overlapped with SDS titrations into buffer between 0 and 2 mM SDS, indicating that CZ does not interact with monomeric SDS (Figure 9A). This correlates well with fluorescence and CD experiments which indicated that no micelles are formed on the surface of CZ and that denaturation only occur when SDS micelles are formed in the bulk phase. At concentrations above 2 mM SDS, an exotermic signal was observed which eventually merged with the buffer signal. The exothermic signal increased with increasing concentration of CZ and the concentration of SDS where the titration merged with the buffer titration increased as well. We define two inflection points: CZ-SDS-1 (the minimum of the exothermic signal) and CZ-SDS-2 (where the titration of CZ merges with the titration of SDS into buffer) (Figure 9B). CZ binds 22.9 ± 1.8 and 34.2 ± 0.9 SDS molecules at the two inflection points (Figure 9C), equivalent to one SDS molecule pr. 12.6 and 8.5 amino acid residues, respectively. The binding of 1 SDS molecule pr. 8.5 amino acid residues at saturation is very low compared to LT and other proteins denatured by SDS. The low binding number may however be due to a combination of several disulfide bonds and the heavy glycosylation of the linker. Glycosylation has previously been shown to decrease the amount of SDS which binds to enzymes (Bagger et al., 2007). In contrast to SDS, CZ titrations with RL overlapped with RL titrations into buffer (Figure 9D). We conclude that CZ does not interact with RL at room temperature.

## Discussion

We undertook this study to compare the impact of the synthetic surfactant SDS and the microbially produced biosurfactant RL on the structure, stability and enzymatic activity of 3 widely used industrial enzymes. Importantly, all three sets of data corroborate each other and demonstrate that SDS displays a great deal of versatility in its type of interaction with the enzymes. One enzyme (SZ) is actually stabilized by SDS while two others are destabilized and denatured but in different ways, LT by SDS clusters formed on the protein below the cmc and CZ by binding of bulk micelles at low stoichiometries. This diversity of binding and unfolding reflects how the enzymes can make specific interactions between protein and SDS. Binding is mediated by a highly concentrated negative charge on the sulfate headgroup in combination with a long and unbranched alkyl chain, promoting binding at multiple different places on the protein surface depending on electrostatic and hydrophobic binding opportunities (Otzen, 2011). For example, two structurally related β-sheet proteins were denatured at sub- and super-cmc SDS concentrations, respectively (Yonath et al., 1977a,b; Nielsen et al., 2007), and we have attributed this to differences in potential electrostatic binding sites. Thus, sub-cmc unfolding may require the presence of cationic hot-spots to attract a multiple number of SDS monomers in a small region of the protein, promoting subsequent cluster formation by association of the adjoining alkyl chains. While we cannot make such simple comparative conclusions for LT vs. CZ as they represent very different structures, we consider it likely that similar mechanisms are at play here. The stabilization of SZ by SDS must reflect the binding of a small number of SDS monomers to one or a few sites on SZ which are found in the native state; simple mass-action then dictates that binding will stabilize the native state rather than denaturing it, just as observed for BSA (Khan et al., 2013).

In contrast to SDS, RL shows a very weak level of interaction with the enzymes, in no case inducing structural changes, at most changing the melting temperature by a few degrees upward or downward and generally having little effect on enzyme activity. For all three enzymes, RL:protein binding stoichiometries are low or undetectable. This does not imply that RL is completely unable to perturb protein structure; we have recently reported that sub-cmc RL is able to denature the notoriously unstable apo-form of α-lactalbumin while super-cmc concentrations are required to denature the disulfide-free protein myoglobin (Andersen and Otzen, 2014b). However, even when denaturing these relatively unstable proteins, the denaturation process is slow and does not involve many binding steps unlike SDS, where efficient denaturation is likely achieved by an accumulation of different binding and denaturation steps. We have attributed the weak binding of RL to a weakly acidic carboxylic head group and a branched hydrophobic chain, both of which promote micelle formation at the expense of (extensive) protein binding (Andersen and Otzen, 2014b). Larger proteins evolved for microbial extracellular secretion are often stabilized against, e.g., proteolytic attack to ensure their ability to persist in an exposed and competitive extracellular environment (Kirk et al., 2002), and this increased stability likely tips the balance against denaturation by RL. Current enzymes used in the detergent industry are typically engineered to withstand denaturation by relatively aggressive mixtures of anionic and nonionic surfactants (Otzen et al., 1999). Thus, RL is likely to be compatible with all the industrial enzymes currently in use in detergents, and may even allow the introduction of enzymes that are sensitive to the present harsh synthetic anionic surfactants. Biosurfactants have already been shown to emulgate vegetable oils efficiently and to be compatible with commercial laundry detergents (Mukherjee, 2007). Thus, there are definitely bright prospects for the inclusion of biosurfactants in future commercial applications.

### Conflict of interest statement

The authors declare that the research was conducted in the absence of any commercial or financial relationships that could be construed as a potential conflict of interest.

## Abbreviations

BS: Biosurfactants
CD: circular dichroism
cmc: critical micelle concentration
CZ: Carezyme
ITC: isothermal titration calorimetry
IP: inflection point
LT: Lecitase
RL: rhamnolipid
SDS: sodium dodecyl sulfate
SZ: Stainzyme.

## References

[B1] Andersen K. K. Oliveira C. L. P. Larsen K. L. Poulsen F. M. Callisen T. H. Westh P.. (2009). The role of decorated SDS micelles in sub-cmc protein denaturation and association. J. Mol. Biol. 391, 207–226. 10.1016/j.jmb.2009.06.019 19523473

[B2] Andersen K. K. Otzen D. E. (2009). How chain length and charge affect surfactant denaturation of acyl coenzyme A binding protein (ACBP). J. Phys. Chem. B 113, 13942–13952. 10.1021/jp905553h 19788195

[B3] Andersen K. K. Otzen D. E. (2014a). Folding of outer membrane protein A in the anionic biosurfactant rhamnolipid. FEBS Lett. 588, 1955–1960. 10.1016/j.febslet.2014.04.004 24735722

[B4] Andersen K. K. Otzen D. E. (2014b). Denaturation of a-lactalbumin and myoglobin by the anionic biosurfactant rhamnolipid. Biochim. Biophys. Acta 1844, 2338–2345. 10.1016/j.bbapap.2014.10.005 25450503

[B5] Andersen K. K. Westh P. Otzen D. E. (2008). A global study of myoglobin-surfactant interactions. Langmuir 15, 399–407. 10.1021/la702890y 18069862

[B6] Bagger H. L. Hoffmann S. V. Fuglsang C. C. Westh P. (2007). Glycoprotein-surfactant interactions: a calorimetric and spectroscopic investigation of the phytase-SDS system. Biophys. Chem. 129, 251–258. 10.1016/j.bpc.2007.06.005 17618035

[B7] Bojsen K. Borch K. Budolfsen G. Fuglsang K. C. Glad S. S. Petri A. (2000). Lipolytic enzyme variants, in PCT International Application WO2000/32758 (Bagsvaerd).

[B8] Chen M. L. Penfold J. Thomas R. K. Smyth T. J. Perfumo A. Marchant R.. (2010). Mixing behavior of the biosurfactant, rhamnolipid, with a conventional anionic surfactant, sodium dodecyl benzene sulfonate. Langmuir 26, 17958–17968. 10.1021/la1031834 21043468

[B9] Daniel H. J. Reuss M. Syldatk C. (1998). Production of sophorolipids in high concentration from deproteinized whey and rapeseed oil in a two stage fed batch process using Candida bombicola ATCC 22214 and Cryptococcus curvatus ATCC 20509. Biotechnol. Lett. 20, 1153–1156 10.1023/A:1005332605003 10077820

[B10] Daverey A. Pakshirajan K. (2010). Kinetics of growth and enhanced sophorolipids production by Candida bombicola using a low-cost fermentative medium. Appl. Biochem. Biotechnol. 160, 2090–2101. 10.1007/s12010-009-8797-3 19834651

[B11] Davies G. J. Dodson G. G. Hubbard R. E. Tolley S. P. Dauter Z. Wilson K. S.. (1993). Structure and function of endoglucanase V. Nature 365, 362–364. 8377830 10.1038/365362a0

[B12] Edwards K. R. Lepo J. E. Lewis M. A. (2003). Toxicity comparison of biosurfactants and synthetic surfactants used in oil spill remediation to two estuarine species. Mar. Pollut. Bull. 46, 1309–1316. 10.1016/S0025-326X(03)00238-8 14550343

[B13] Franzetti A. Tamburini E. Banat I. M. (2010). Applications of biological surface active compounds in remediation technologies. Adv. Exp. Med. Biol. 672, 121–134. 10.1007/978-1-4419-5979-9_9 20545278

[B14] Jones M. N. Manley P. Midgley P. J. Wilkinson A. E. (1982b). Dissociation of bovine and bacterial catalases by sodium n-dodecyl sulfate. Biopolymers 21, 1435–1450. 7115898 10.1002/bip.360210712

[B15] Jones M. N. Manley P. Wilkinson A. (1982a). The dissociation of glucose oxidase by sodium n-dodecyl sulphate. Biochem. J. 203, 285–291. 7103941 10.1042/bj2030285 PMC1158221

[B16] Jönsson B. Lindman B. Holmberg K. Kronberg B. (1998). Surfactants and Polymers in Aqueous Solutions. New York, NY: Wiley & Sons.

[B17] Kalyanasundaram K. Thomas J. K. (1977). Environmental effects on vibronic band intensities in pyrene monomer fluorescence and their application in studies of micellar systems. J Am. Chem. Soc. 99, 2039–2044 10.1021/ja00449a004

[B18] Khan J. M. Chaturvedi S. K. Khan R. H. (2013). Elucidating the mode of action of urea on mammalian serum albumins and protective effect of sodium dodecyl sulfate. Biochem. Biophys. Res. Commun. 441, 681–688. 10.1016/j.bbrc.2013.10.055 24394939

[B19] Kirk O. Borchert T. V. Fuglsang C. C. (2002). Industrial enzyme applications. Curr. Opin. Biotechnol. 13, 345–351. 10.1016/S0958-1669(02)00328-2 12323357

[B20] Lima T. M. Procopio L. C. Brandao F. D. Carvalho A. M. Totola M. R. Borges A. C. (2011). Biodegradability of bacterial surfactants. Biodegradation 22, 585–592. 10.1007/s10532-010-9431-3 21053055

[B21] Liu J. Shi J. Li J. Yuan X. (2011). Characterization of the interaction between surfactants and enzymes by fluorescence probe. Enzyme Microb. Technol. 49, 360–365. 10.1016/j.enzmictec.2011.06.014 22112561

[B22] Martinelle M. Holmquist M. Hult K. (1995). On the interfacial activation of Candida antarctica lipase A and B as compared with Humicola lanuginosa lipase. Biochim. Biophys. Acta 1258, 272–276. 10.1016/0005-2760(95)00131-U 7548197

[B23] Mogensen J. E. Sehgal P. Otzen D. E. (2005). Activation, inhibition, and destabilization of Thermomyces lanuginosus lipase by detergents. Biochemistry 44, 1719–1730. 10.1021/bi0479757 15683256

[B24] Mukherjee A. K. (2007). Potential application of cyclic lipopeptide biosurfactants produced by Bacillus subtilis strains in laundry detergent formulations. Lett. Appl. Microbiol. 45, 330–335. 10.1111/j.1472-765X.2007.02197.x 17718848

[B25] Nelson C. A. (1971). The binding of detergents to proteins. I. The maximum amount of dodecyl sulfate bound to proteins and the resistance to binding of several proteins. J. Biol. Chem. 246, 3895–3901. 4327191

[B26] Nielsen A. D. Arleth L. Westh P. (2005). Interactions of Humicola insolens cutinase with an anionic surfactant studied by small-angle neutron scattering and isothermal titration calorimetry. Langmuir 21, 4299–4307. 10.1021/la047299+ 16032839

[B27] Nielsen M. M. Andersen K. K. Westh P. Otzen D. E. (2007). Unfolding of b-sheet proteins in SDS. Biophys. J. 92, 3674–3685. 10.1529/biophysj.106.101238 17351005 PMC1853130

[B28] Otzen D. (2011). Protein-surfactant interactions: a tale of many states. Biochim. Biophys. Acta 1814, 562–591. 10.1016/j.bbapap.2011.03.003 21397738

[B29] Otzen D. E. Christiansen L. Schülein M. (1999). A comparative study of the unfolding of the endoglucanase Cel45 from *Humicola insolens* in denaturant and surfactant. Prot. Sci. 8, 1878–1887. 10.1110/ps.8.9.1878 10493589 PMC2144393

[B30] Otzen D. E. Sehgal P. Westh P. (2009). a-lactalbumin is unfolded by all classes of detergents but with different mechanisms. J. Coll. Int. Sci. 329, 273–283. 10.1016/j.jcis.2008.10.021 18977000

[B31] Patel M. (2003). Surfactants based on renewable raw materials. J. Ind. Ecol. 7, 47–62 10.1162/108819803323059398

[B32] Pitt-Rivers R. Impiombato F. (1968). Binding of sodium dodecyl sulphate to various proteins. Biochem. J. 109, 825–830. 4177067 10.1042/bj1090825 PMC1187034

[B33] Reynolds J. A. Tanford C. (1970). Binding of dodecyl sulfate to proteins at high binding ratios. Possible implications for the state of proteins in biological membranes. Proc. Natl. Acad. Sci. U. S.A. 66, 1002–1005. 10.1073/pnas.66.3.1002 5269225 PMC283150

[B34] Sanchez M. Aranda F. J. Espuny M. J. Marques A. Teruel J. A. Manresa A.. (2007). Aggregation behaviour of a dirhamnolipid biosurfactant secreted by Pseudomonas aeruginosa in aqueous media. J. Colloid Interface Sci. 307, 246–253. 10.1016/j.jcis.2006.11.041 17182054

[B35] Sanchez M. Aranda F. J. Espuny M. J. Marques A. Teruel J. A. Manresa A.. (2008). Thermodynamic and structural changes associated with the interaction of a dirhamnolipid biosurfactant with bovine serum albumin. Langmuir 24, 6487–6495. 10.1021/la800636s 18481884

[B35a] Schülein M. (1997). Enzymatic properties of cellulases from Humicola insolens. J Biotechnol. 57, 71–81. 9335167 10.1016/s0168-1656(97)00090-4

[B36] Smith L. A. Hammond R. B. Roberts K. J. Machin D. McLeod G. (2000). Determination of the crystal structure of anhydrous sodium dodecyl sulphate using a combination of synchrotron radiation powder diffraction and molecular modelling techniques. J. Mol. Struct. 554, 173–182 10.1016/S0022-2860(00)00666-9

[B37] Tanford C. (1980). The Hydrophobic Effect. Formation of Micelles and Biological Membranes, 2nd Edn. New York, NY: Wiley & Sons.

[B38] Wang L. L. Wang Y. Hu C. Y. Cao Q. Yang X. H. Zhao M. M. (2011). Preparation of diacylglycerol-enriched oil from free fatty acids using lecitase ultra-catalyzed esterification. J. Am. Oil Chem. Soc. 88, 1557–1565 10.1007/s11746-011-1821-0

[B39] Yonath A. Podjarny A. Honig B. Sielecki A. Traub W. (1977a). Crystallographic studies of protein denaturation and renaturation. 2. Sodium dodecyl sulfate induced structural changes in triclinic lysozyme. Biochemistry 16, 1418–1424. 849424 10.1021/bi00626a028

[B40] Yonath A. Sielecki A. Moult J. Podjarny A. Traub W. (1977b). Crystallographic studies of protein denaturation and renaturation. 1. Effects of denaturants on volume and X-ray pattern of cross-linked triclinic lysozyme crystals. Biochemistry 16, 1413–1417. 557340 10.1021/bi00626a027

